# Decrypting the Thalamic Subnuclei and Functional Composites in Adolescents With Psychotic Experiences

**DOI:** 10.1192/bjo.2024.234

**Published:** 2024-08-01

**Authors:** Sahar Riaz, Michael O'Connor, Anurag Nasa, Mary Cannon, Darren Roddy

**Affiliations:** Royal College of Surgeons in Ireland, Dublin, Ireland

## Abstract

**Aims:**

The thalamus, a dual grey matter formation within the diencephalon is thought to be involved in psychosis. It consists of distinct nuclei with specific functions. To date no study has investigated the volumes of the thalamic nuclei in young adolescents with Psychotic Experiences (PEs).

**Methods:**

This study used T1 imaging with Freesurfer analysis to investigate the differences in thalamic nuclei in 98 young people (53 with PEs) over three time points, from ages 11 to 18. A linear mixed effects (LME) model was used to examine the longitudinal nature of the data.

**Results:**

The findings were entirely left sided – specifically a smaller left whole thalamus (p = 0.04), significant reduction in the size of the left pulvinar (p = 0.008) and a slight increase in the size of left ventral nucleus (p = 0.005).

**Conclusion:**

This study found significant volumetric differences in thalamic functional composite nuclei between adolescents with a history of PE compared with healthy controls. Two such nuclear groups survived post-hoc DTR testing, the left ventral and left pulvinar nuclei. The pulvinar nucleus demonstrated a reduced volume over time in PE groups compared with healthy controls whilst the left ventral nucleus demonstrated an increased volume over time in PE groups compared with controls. The thalamus has been shown to be actively involved in the modulation of cortico-cortical communication via cortico-thalamo-cortical pathways, thus synchronizing the activity of the cortex during tasks that require attention. One of the core deficits believed to be a part of psychotic illnesses is the inappropriate modulation of attention through various cortical networks. This disrupted modulation results in a lack of control of goal-directed behaviour and can be attributed to the changes seen in pulvinar in psychotic illnesses, thus resulting in impairment in the integrity of sensory information and context processing. The affiliation of the ventral thalamic nucleus to the dopaminergic system, particularly the substantia nigra, may aid in explaining why this nucleus demonstrates larger volumes in adolescents with PEs compared with healthy controls over time.

More research needs to be done on following this cohort up, specifically investigating changes in thalamic nuclei in those who develop a diagnosable psychotic disorder.

